# WHICH MEASURES OF STRESS ARE ASSOCIATED WITH ACCELERATED BIOLOGICAL AGING?

**DOI:** 10.1093/geroni/igad104.1962

**Published:** 2023-12-21

**Authors:** Kyle Bourassa, Avshalom Caspi, Terrie Moffitt

**Affiliations:** Durham VA Medical Center, Durham, North Carolina, United States; Duke University, Durham, North Carolina, United States; Duke University, Durham, North Carolina, United States

## Abstract

Stress and stressful events are associated with poorer health. There are multiple approaches to measure stress—from assessing subjective perceptions of stress to assessing the development of posttraumatic stress disorder (PTSD)—and multiple periods that can be assessed across the lifespan. How might stress result in poorer health? One plausible physiological mechanism is accelerated biological aging, which precedes many poor health outcomes. Our study tested which measures of stress were associated with accelerated biological aging in adulthood using 955 participants from the Dunedin Longitudinal Study. The measures of stress included perceived stress, stressful life events, adverse childhood experiences (ACEs), and PTSD assessed between ages 32 to 45 years and accelerated biological aging in midlife was measured using the Pace of Aging. Higher levels of all four stress measures were significantly associated with accelerated aging in separate models (all βs > 0.14, all ps < .001). In a combined model, more perceived stress and more stressful life events remained associated with faster aging and together the four stress measures accounted for 7% of the variance in biological aging. The magnitudes of the associations between the four measures of stress and biological aging were comparable to associations for smoking and low education, two established risk factors for accelerated aging. Assessing stress, particularly perceived stress, could help identify people at risk of accelerated aging. Stress perceptions could be a potentially modifiable treatment target to slow the rate at which people are aging, with the goal of improving health as people age.

